# The effect of electrical muscle stimulation on intentional binding and explicit sense of agency

**DOI:** 10.7717/peerj.17977

**Published:** 2024-09-19

**Authors:** Miwa Nagai, Kazuhiro Matsui, Keita Atsuumi, Kazuhiro Taniguchi, Hiroaki Hirai, Atsushi Nishikawa

**Affiliations:** 1 Graduate School of Engineering Science, Osaka University, Toyonaka, Japan; 2 Graduate School of Information Sciences, Hiroshima City University, Hiroshima, Japan; 3 Faculty of Human Ecology, Yasuda Women’s University, Hiroshima, Japan

**Keywords:** Intentional binding, Sense of agency, Electrical muscle stimulation

## Abstract

The motivating question for this study is determining whether electrical muscle stimulation (EMS)-induced movements can extend the user’s ability without reducing the sense of agency. Moreover, it is crucial to find the timing of the EMS application that is robust against individual differences and environmental changes. Previous studies have reported that the user-specific EMS-application timings, determined through explicit measures of sense of agency, would effectively shorten their reaction time in a push task while maintaining their sense of agency. However, no study has investigated EMS-application timings in relation to implicit measures of sense of agency. Intentional binding, an example of an implicit measure, refers to the phenomenon whereby the interval between an intentional action and the subsequent perceptual outcome is typically perceived to be shorter than the actual interval. By measuring this perceptual shift using a Libet clock, we have identified an EMS-application timing that accelerates the users’ push action while maintaining their sense of agency. First, to conduct the EMS-application experiment while appropriately maintaining the intentional binding effect, we designed a new push task such that a pre-action, as the base timing of the EMS-application trigger, always occurs just before the push movement. (1) We showed the difference between the action-binding effect of EMS-induced involuntary movements and voluntary push movements. Subsequently, (2) we identified the EMS application timing that significantly shifted judgments of action tasks while accelerating voluntary movements. Additionally, (3) we demonstrated that the EMS application could accelerate user pushing movement while maintaining the sense of agency at this specific application time. The proposed EMS in the novel pushing setup was found to be robustly effective against individual and environmental changes.

## Introduction

Electrical muscle stimulation (EMS) has advantages over the exoskeletal device application in that it can teach which muscles to activate and to what degree (Lopes & Baudisch, 2013; Lopes et al., 2015; Niijima et al., 2021). Thus, it is considered valuable as a motor-learning support method. However, if EMS forces the user to perform movements beyond their motor abilities, the user will frequently feel that they are not in control of the movement, *i.e*., their sense of agency will become impaired, which may limit motor adaptation and learning (Takahashi et al., 2012; Gandolla et al., 2016). This augmentation of action skills, achieved through the close integration of humans and computers acting autonomously with each other (referred to as human–computer integration; HInt (Farooq & Grudin, 2016)), requires the user to perceive themselves as an agent (Moore, 2016; Cornelio et al., 2022).

Studies have demonstrated that user-specific EMS-application timing, based on prior measurements of reaction times and self-reported sense of agency of participants, would reduce their reaction time while maintaining their sense of agency (Kasahara, Nishida & Lopes (2019): CHI conference proceedings, Tajima et al. (2022)). Kasahara et al. (2021) demonstrated that in cases where EMS was applied while preserving the user’s sense of agency, the user could maintain an accelerated reaction even after EMS removal.

There are two methods for measuring the sense of agency: explicit measures (such as questionnaires using a Likert scale) and implicit measures (which indirectly measure the perceptual changes associated with the sense of agency). The former explicit measure is widely used typical and simple method. However, it has the limitation of judgment bias, where healthy participants misattribute to their outcomes that deviate strongly from their actual actions (Franck et al., 2001) or positive outcomes with improved performance (Wegner, 2003; Sato & Yasuda, 2005). An example of the latter is intentional binding, which is a phenomenon wherein more intentional behavior results in a shorter perceived time interval between the intentional movement and subsequent outcome because of the perceived temporal attraction between the action and the effect (Haggard, Clark & Kalogeras, 2002; Moore & Obhi, 2012). Researchers have posited that this implicit measure could be used as a relatively objective measure of the sense of agency without requiring an explicit judgment of the agency (Engbert, Wohlschläger & Haggard, 2008; Ebert & Wegner, 2010). Additionally, recent studies in the field of human–computer interaction have evaluated device usability and interaction quality by measuring intentional binding (Coyle et al., 2012). Notably, however, in some studies, the intentional binding does not correlate with the self-reported sense of agency (Cravo, Claessens & Baldo, 2009; Obhi & Hall, 2011; Dewey & Knoblich, 2014). In addition, it has been previously reported that intentional binding effects equivalent to voluntary movements occur for both mechanical actions (Buehner, 2012) and a virtual hand (Suzuki et al., 2019). The limitations of implicit and explicit measures are that they measure only partial (and partially overlapping) aspects of the agency experience: the feeling of agency and judgment of agency (Synofzik, Vosgerau & Newen, 2008). Therefore, we expected that their combination will complement each other and be useful in measuring the effects of small changes in the offset of the EMS application on the sense of agency.

In this article, we propose an EMS application method that combines the acceleration of movement through an EMS-induced task with the maintenance of the sense of agency. (1) A novel switch-pushing system is designed so that a movement available as an EMS trigger occurs before each push. (2) The optimal offset for the EMS application is determined based on intentional binding measures, which is unprecedented in EMS experiments.

The specific switch-pushing system designed in this study was proposed to integrate the intentional binding with accelerated EMS application. In previous accelerated EMS experiments (Kasahara, Nishida & Lopes, 2019), visual stimuli were employed as cues for movement initiation and triggering to apply EMS prior to voluntary movements. Contrarily, in the intentional binding experiments, participants must determine the onset of the movement themselves. We proposed a new switch-pushing design that resolves this discrepancy between these two experiments. Participants were required to first hold down the switch (switch-on), and when they wished to operate the switch, they would release their finger from the switch (switch-off) and push it again (switch-on). They performed these actions sequentially as quickly as possible. Using the switch-off action, which always occurred at least several tens of milliseconds before the switch was pushed, as a trigger for the EMS application, the EMS could be applied before the voluntary push while the participants determined the onset time of the action. Previous studies found that physical effort is positively associated with both explicit self-attributions (Minohara et al., 2016) and intentional binding effects (Demanet et al., 2013). However, minimal effort (0.5–2.0 N) is required to press the switch used in this experiment. Therefore, we assume that the wait state of holding down the switch in the proposed voluntary push system is approximately equivalent to that of not holding down the switch in the traditional push system used in intentional binding experiments. Thus, the intentional binding effect of the proposed voluntary push system could be appropriately maintained. Unlike visual stimuli that were not directly related to the user’s intention, the proposed voluntary push method was directly related to the user’s intention. Therefore, using this as a basis for EMS triggering, the EMS application might be robust even when the task difficulty or the user’s attention changes.

Three hypotheses were constructed to verify the effectiveness of the proposed application method.

**Hypothesis 1**: The difference in the intentional binding effects makes it possible to distinguish the EMS-induced movements as involuntary movements and the proposed switch-pushing action as a voluntary movement.

**Hypothesis 2**: Optimal EMS application timing can be determined to sustain the intentional binding effect by measuring changes in this effect across different EMS application times.

**Hypothesis 3**: The movement induced by the EMS applied at the time identified by the intentional binding measurement can accelerate the switch-pushing action while maintaining the user’s sense of agency.

Each of these three hypotheses was experimentally tested in this study (Table 1).

**Table 1:** Experimental structure.

Experiments	Experiment 1	Experiment 2	Experiment 3
Goals	Distinguish between the proposed voluntary push and involuntary EMS push	Determine the optimal EMS application time from the binding effect	Evaluate the timing of the identified EMS application
Tasks	Intentional binding measurement	Intentional binding and self-reported sense of agency measurement	Reaction time task
Findings	The presence or absence of a significant action-binding effect distinguishes voluntary push from involuntary EMS push	Among six EMS timings, a timing was found that significantly maintained the action binding	The EMS timing identified in Experiment 2 accelerated the pushing movement while maintaining the sense of agency

## Experiment 1: measurements of the intentional bindings for the ems-induced movement and the proposed voluntary push movement

This experiment was conducted to confirm that the measure of intentional binding can be used to discriminate voluntary movements from involuntary movements produced by EMS.

### Methods

#### Participants

Forty-eight healthy, right-handed volunteers (thirteen females; mean age = 21.9 years; S.D. = 2.7 years) participated in the experiment. The sample size was determined to be a minimum of 43 participants based on a pilot study with a gross number of eight participants, ensuring that the power of the test for detecting the difference between voluntary proposed pushing and involuntary EMS pushing exceeded 0.8. All participants were provided with written informed consent following the procedures approved by the Ethics Committee at the Osaka University Graduate School of Engineering Science (approval number: R4-12). All of them were unaware of the experiment’s purpose and intentional binding; this was their first experience with EMS.

#### System

The Libet clock driven by a stepper motor (120 steps per revolution) was employed. Referring to a previous study (Haggard, Clark & Kalogeras, 2002), the clock had a single hand rotating clockwise, with a period of 2,560 ms, and a dial with 60 scales and numerical values each separated by an interval of five scales. The position of the clock hand was identified by detecting the start point using a photoreflector.

**Figure 1:**
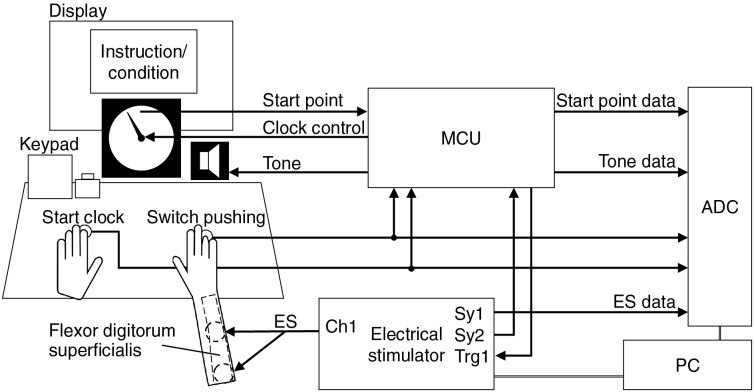
Experimental setup. Image created in Microsoft PowerPoint. Icons taken from Microsoft PowerPoint.

Figure 1 shows the experimental system configuration. The participants pushed the right-hand switch as the voluntary or EMS-induced movement task and the left-hand switch to start the clock. Both switches were optical switches that could be turned on by pressing 1.0 $\pm$ 0.5 mm with a force of 0.5–2.0 N. Thus, they could be pushed by the ring finger. The microcontroller (PIC16F1827) triggered the electrical stimulation device and controlled the tone (explained in the *Tasks* part) and the clock. An analog-to-digital converter (AI-1608AY-USB, Contec, Melbourne, FL, USA) was employed to acquire the data from the clock start point, two switches, electrical stimulation, and tone at a sampling rate of 1 kHz.

#### Tasks

The participants were asked to judge the onset time of the task they were instructed to perform. Under the single-task baseline condition, we set up the following four tasks:

***Voluntary*:** Participants first held the switch down using their right ring finger, then released their finger from the switch (switch-off) and pushed it again (switch-on) at an arbitrary time and judged the onset time of the switch-on movements. However, they were instructed to avoid responding to a specific tick position as a target or during the first rotation of the clock hand.

***Involuntary EMS*:** EMS was applied randomly, resulting in the flexion of the right ring finger. Participants then judged the completion time of the involuntary switch-pushing movement.

***Sham EMS*:** Sham EMS, which produces a sensation of stimulation but no actuation, was applied randomly. The participants reported the time at which they felt the stimulation.

***Tone*:** Participants were asked to judge the time of a randomly applied tone of 1 kHz frequency for 100 ms.

Random stimulation and tone were delivered between 2.5 and 7.6 s after the start of the trial.

Under the operant condition, three tasks, excluding the single-tone task, were followed by a tone (frequency = 1 kHz and duration = 100 ms) from a loudspeaker 250 ms later. The reference time for the tone offset was the time the switch was pushed in the *Voluntary* and *Involuntary EMS* conditions, as well as the time the electrical stimulation was applied in the *Sham EMS* condition since the switch was not pushed. The participants were asked to judge the onset time of the action or the tone for each task.

**Figure 2:**
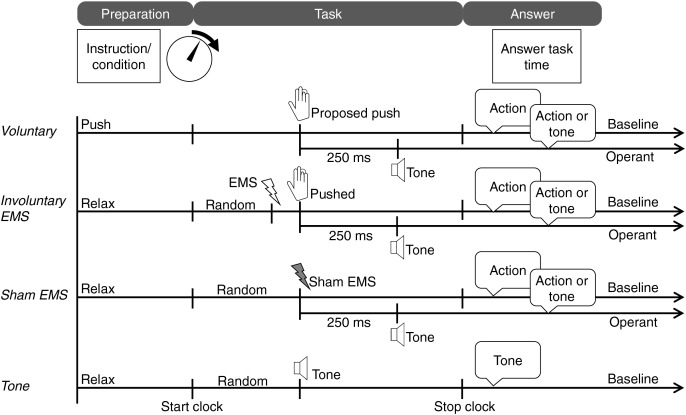
Tasks of Experiment 1. Image created in Microsoft PowerPoint. Icons taken from Microsoft PowerPoint.

In total, there were 10 blocks for this experiment, including four baseline tasks and three operant tasks with two response types (action or tone), as shown in Fig. 2. Each participant performed the 10 blocks in a different random order. There were 20 trials in each block, for a total of 200 trials.

#### Procedure

First, the participants read instructions on the display of the condition in which the electrical stimulation or the tone was given (or not) and instructions regarding what time to judge, and what state the participant’s right ring finger should be in while waiting for the task to begin. The finger-waiting condition was an addition to the instructions in the previous study (Haggard, Clark & Kalogeras, 2002). In the *Voluntary* condition, the participants held down the switch with their right ring finger, whereas in the other conditions, they did not push the switch, but waited with their fingers above the switch so that the movement caused by the EMS could push the switch. They started the clock by pushing the left-hand switch as the initiation of the designated trial. Participants entered the judged time using a numeric keypad with their left hand after each trial. We encouraged them to respond by reading the time from the clock as accurately as possible. The clock was randomly stopped 1.5–2.52 s after the task was completed.

#### Parameters of the EMS and sham EMS

For the EMS tasks, a pulsed wave was used with the following parameters: frequency = 100 Hz, width = 800 $\rm\mu s$, number of pulses = 3, and pulse amplitude = 2.5–16.0 mA (based on a study by Kasahara, Nishida & Lopes (2019)). Herein, the target muscle was the flexor digitorum superficialis, and two electrodes were placed across its motor point, as described by Bao et al. (2018). By inducing flexion of all finger joints (except the DIP joint) of the ring finger, the EMS confirmed that the target muscles were stimulated. For each participant, these parameters and a current value were set so that only the ring finger was flexed and the other fingers had minimal or no movement. Seven participants could not flex the ring finger independently with EMS; thus, they used the middle finger (five participants) or little finger (two participants) as an alternative. For the sham EMS with a 1,000 Hz pulse wave, the pulse width, number of pulses, and pulse amplitude were exploratively adjusted to 50–500 $\rm\mu s$, 30–120, and 3.5–16.5 mA, respectively, to ensure stimulation without finger movement. The same position of the electrodes was used for the EMS and the sham EMS. These stimulations were generated by STG4002 (Multichannel systems MCS GmbH) and triggered using a microcontroller. For emergencies, a safety switch was placed near the participants to shut off the stimulator.

### Results and discussion

**Figure 3:**
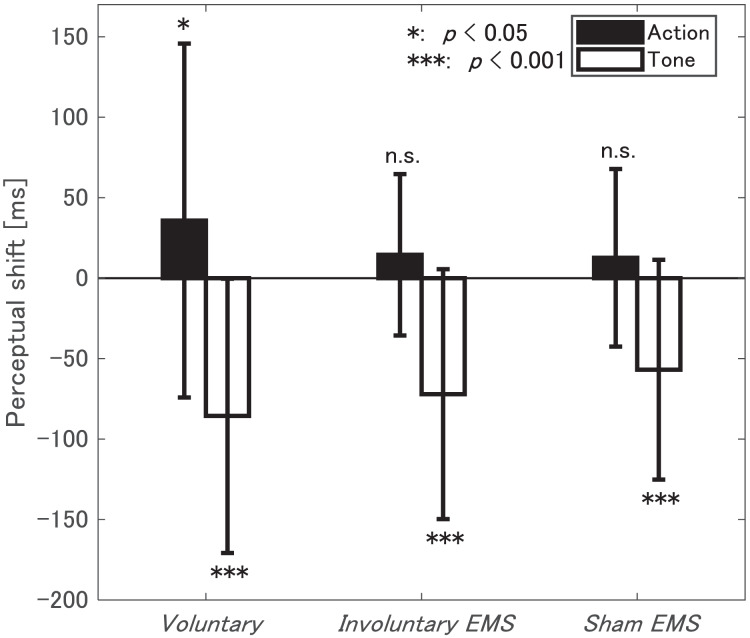
Mean and SD of perceptual shift for action and tone judgments.

The judgment error, which indicates the difference between a participant’s judged time and the actual time for the given task, was calculated for each trial. A negative judgment error signifies predictive judgment, while a positive judgment error indicates delayed judgment. Based on previous studies (Strother, House & Obhi, 2010; Obhi & Hall, 2011), trials were excluded from analysis if judgment errors exceeded or fell below three standard deviations from the mean judgment error for each participant. Additionally, trials where the time between EMS and pushing—defined as the EMS actuation time—exceeded 200 ms in the *Involuntary EMS* condition, were excluded because the stimulus did not contribute to the pushing movement. These combined criteria resulted in the removal of 1.13% of the data. The change in time perception due to the additional tone task in the operant condition was calculated as the perceptual shift for each condition: the judgment error for the baseline condition was subtracted from the judgment error for the operant condition. A positive (negative) perceptual shift in the action task indicates a bound (unbound) state, while a positive (negative) perceptual shift in the tone task indicates an unbound (bound) state. The absolute value of the perceptual shift represents the magnitude of binding/unbinding. Judgment errors and perceptual shifts are presented in Table 2 and Fig. 3.

**Table 2:** Judgment error, perceptual shift, and paired t-test results between baseline and operant conditions in Experiment 1.

Condition	Judgment event	Mean judgment error (S.D.) (ms)		Mean perceptual shift (S.D.) (ms)
		Baseline	Operant	
*Voluntary*	Action	−21 (135)	15 (100)	36 (110), $t(47) = 2.254$; $p = 0.0289$
	Tone		−102 (144)	−86 (85), $t(47) = 6.958$; $p\; < \; 0.0001$
*Involuntary EMS*	Action	5 (94)	20 (81)	13 (55), $t(47) = 2.009$; $p = 0.0503$
	Tone		−89 (108)	−57 (68), $t(47) = 6.432$; $p\; < \; 0.0001$
*Sham EMS*	Action	23 (107)	36 (98)	15 (50), $t(47) = 1.586$; $p = 0.1195$
	Tone		−74 (94)	−72 (78), $t(47) = 5.775$; $p\; < \; 0.0001$
*Tone*	Tone	−17 (68)		

To distinguish between involuntary EMS and voluntary movements, we simply assessed whether the perceptual shift was significant in each condition. In particular, we examined whether we could reject the hypothesis that judgment errors were similar between baseline and operant conditions using a paired t-test. For the proposed voluntary push in the *Voluntary* condition, there was a significant later shift in action judgments ( $p = 0.0289$) and a significant early shift in tone judgments ( $p\; < \; 0.0001$). In the two conditions without voluntary movements, the *Involuntary EMS* and *Sham EMS* conditions, there was a significant early shift in tone judgments ( $p\; < \; 0.0001$) but no significant shift in action judgments ( $p = 0.0503$ in *Involuntary EMS* and $p = 0.1195$ in *Sham EMS*). Here, the nonsignificant shifts in action judgments during EMS-induced involuntary pushes ( $p = 0.0503$) were supported by sufficiently small effect sizes (Cohen’s $d = 0.1659$), whereas the significant shifts during voluntary pushes ( $p = 0.0289$) were supported by not small effect sizes ( $d = 0.3008$).

We have reproduced a typical intentional binding effect for the proposed method of voluntary pushing (Haggard, Clark & Kalogeras, 2002). For involuntary EMS movements, the significant shift of the tone judgments is consistent with the results of previous studies wherein involuntary movements cause subsequent outcomes, *e.g*., observing the movements of others with the intention of joint action (Strother, House & Obhi, 2010; Obhi & Hall, 2011), mechanical involuntary movements in which the participants are encouraged to attribute the movement to themselves (Dogge et al., 2012), and finger movements pushed down by a machine (Buehner, 2015). Participants in the *Involuntary EMS* condition perceived the same causality between the action and tone as in the *Voluntary* condition because the tone is triggered by the ring finger pushing the same switch. In other words, if EMS failed to push the switch, the experimenter instructed the participant to retry the trial, similar to a failed voluntary push. This shared causality was designed to combine voluntary pushes and involuntary EMS pushes in the next experiment to determine the optimal EMS application time. Thus, the results suggest that the difference in intentionality between EMS-induced and voluntary movements can be distinguished by a significant shift in the action judgment. Causality between action and subsequent outcome alone is insufficient for the action-binding effect; causality and intentionality are necessary conditions (Cravo, Claessens & Baldo, 2009, 2011). The tasks in the *Sham EMS* condition lacked intentionality or causality, because the tactile sensation of the sham EMS is not an intentional task, and the sham EMS and subsequent tones are triggered by a microcontroller. However, tone judgments were significantly shifted, contrary to the minimal or repulsive shifts reported in two temporally contiguous simple sensory tasks (Haggard, Clark & Kalogeras, 2002; Buehner, 2015). It is possible that causality was mislearned due to the temporal priority, contiguity, and constant conjunction of the action and tone tasks. However, this explanation alone is insufficient to account for the strong tone binding observed in all conditions (including the subsequent experiment), and the influence of the experimental design itself should be considered (see Discussion section for details).

The purpose of this experiment was to distinguish between involuntary EMS and voluntary movements using an implicit sense of agency index, namely intentional binding. We found that a significant shift in the action task clearly distinguished voluntary from involuntary EMS movements and sham EMS sensations. We infer from the experimental conditions that this distinction reflects differences in the intentionality of action tasks rather than the causality between action and tone. In the next experiment, we investigated the modulation of perceptual shifts, especially in action judgments in combinations of involuntary EMS and voluntary movements triggered in stages.

## Experiment 2: intentional binding effect at different ems application times

The purpose of this experiment was to reveal the changes in the intentional binding effect with the EMS application time during voluntary movements and to determine the optimal EMS application time from the results. The results of Experiment 1 showed the respective intentional binding effects for the *Voluntary*, *Involuntary EMS*, and *Sham EMS* conditions. In Experiment 2, the combinations of these conditions, *i.e*., EMS or sham EMS accompanying voluntary movement, were set up to enhance or inhibit the intentional binding effect. In addition, the sense of agency of the proposed voluntary push movement with the EMS applied in stepwise time was explicitly measured and compared with the results for implicit measures.

### Method

#### Participants

Seventeen healthy right-handed participants (eight females; mean age = 23.1 years; S.D. = 9.3 years) were recruited for the experiment. The sample size was determined based on a previous study (Haggard & Clark, 2003). None of them had participated in Experiment 1. All participants were provided with written informed consent following the procedures approved by the Ethics Committee at the Osaka University Graduate School of Engineering Science (approval number: R4-12). All of them were unaware of the experiment’s purpose and intentional binding; this was their first experience with EMS.

#### Tasks and procedure

**Figure 4:**
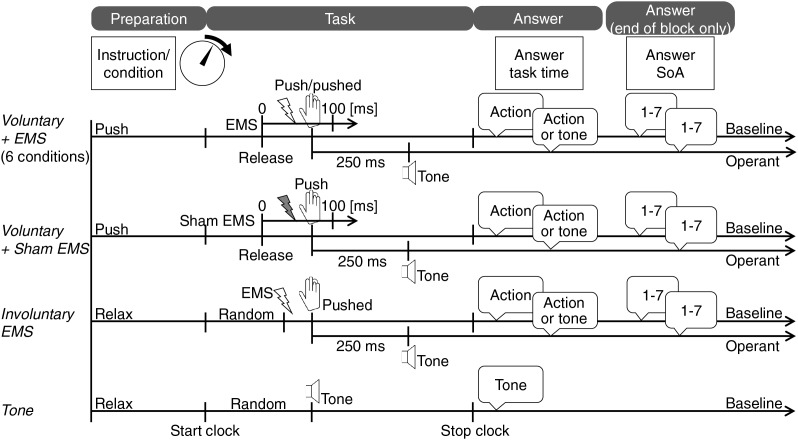
Tasks of Experiment 2. Image created in Microsoft PowerPoint. Icons taken from Microsoft PowerPoint.

We employed an extension of the task in Experiment 1 (Fig. 4) to measure two effects: the intentional binding effect based on time judgments and the sense of agency using a questionnaire.

Nine tasks were incorporated in the baseline condition.

***Voluntary + EMS*:** The participants were instructed to perform the proposed voluntary push action at an arbitrary time. EMS was applied 0–100 ms (20-ms intervals; six conditions) after the finger was released the switch. For the proposed voluntary push movement in the *Voluntary* condition in Experiment 1, the mean pushing time from switch-off to switch-on was 123.3 (S.D. = 82.16) ms. Thus, all EMS applications likely occurred before voluntary pushing. The participants were asked to judge when the switch was first pushed. That is, they were asked to judge the time at which they had pushed the switch if they felt that the voluntary push movements were faster, and to judge the time at which the EMS had pushed the switch if they felt that the EMS-induced push movements were faster.

***Voluntary + Sham EMS*:** The participants pushed the switch with the proposed method and judged the time achieved. After a random time delay (0–100 ms) from the switch-off time, sham EMS without induced movement was applied. This condition was set as the voluntary control condition.

***Involuntary EMS*:** EMS was applied randomly similar to the case in Experiment 1, inducing the flexion of the right ring finger. The participants judged the completion time of the involuntary pushing action.

***Tone*:** The participants were asked to judge the time of a randomly applied tone, the same as in Experiment 1.

Eight tasks, excluding the tone task, were incorporated in the operant condition. The first switch push was followed by an auditory tone 250 ms later, similar to the case in Experiment 1.

Twenty-five blocks were randomly selected, including nine baseline and eight operant tasks with two response types. There were 15 trials in each block, yielding total 375 trials. To reduce the burden on participants and minimize the difference in trial counts between Experiments 1 and 2, we reduced the number of trials per block to about $2/3$ of Experiment 1 based on previous studies (Buehner, 2012; Beck, Di Costa & Haggard, 2017). At the end of each block, participants rated their sense of agency for the movement that first achieved switch pushing according to the following seven-point Likert scale: “I strongly believe that I caused the pushing.” = 7 from “I strongly believe that I didn’t cause the pushing.” = 1.

The experimental system used in Experiment 1 was applied. As in the previous experiment, the parameters of the EMS and the sham EMS were calibrated for the participants. Four participants could not flex the ring finger independently with EMS; thus, they used the middle finger as an alternative.

### Results and discussion

We excluded trials using the same outlier criteria as in Experiment 1. In particular, trials were removed if the EMS actuation times exceeded 200 ms or if the judgment errors fell outside the range of $\mu \pm 3\sigma$ for each participant. This resulted in the removal of 2.23% of the data.

**Figure 5:**
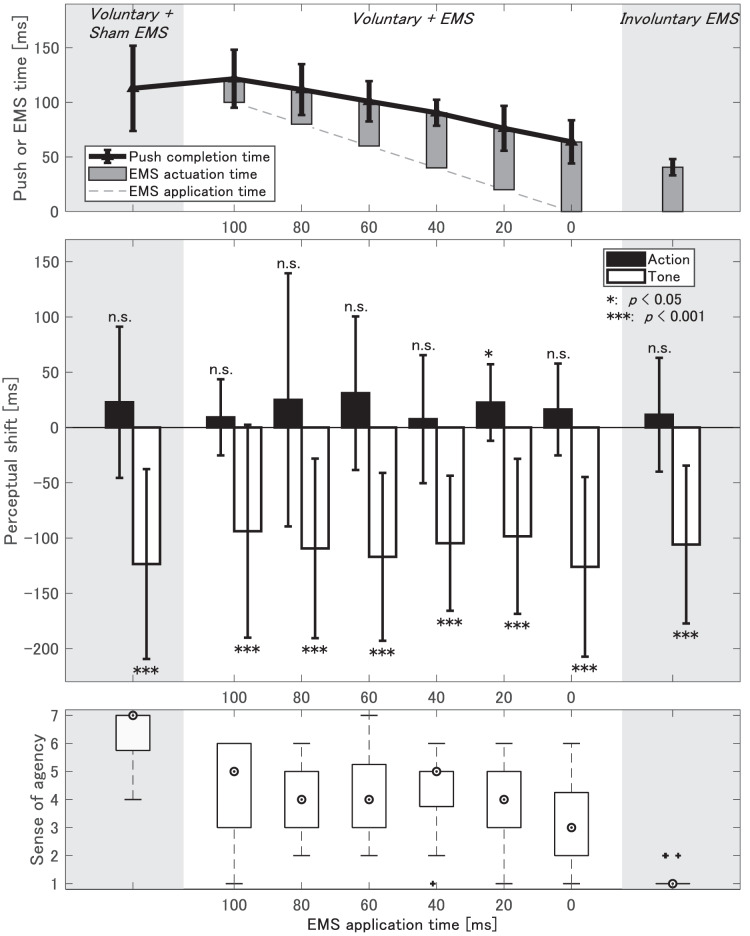
Results of Experiment 2. Top, Mean and S.D. of the time from switch release or EMS application to switch pushing action (push completion time and EMS actuation time, respectively); middle, Mean and S.D. of perceptual shift for action and tone judgments; bottom, Median of self-reported sense of agency.

**Figure 6:**
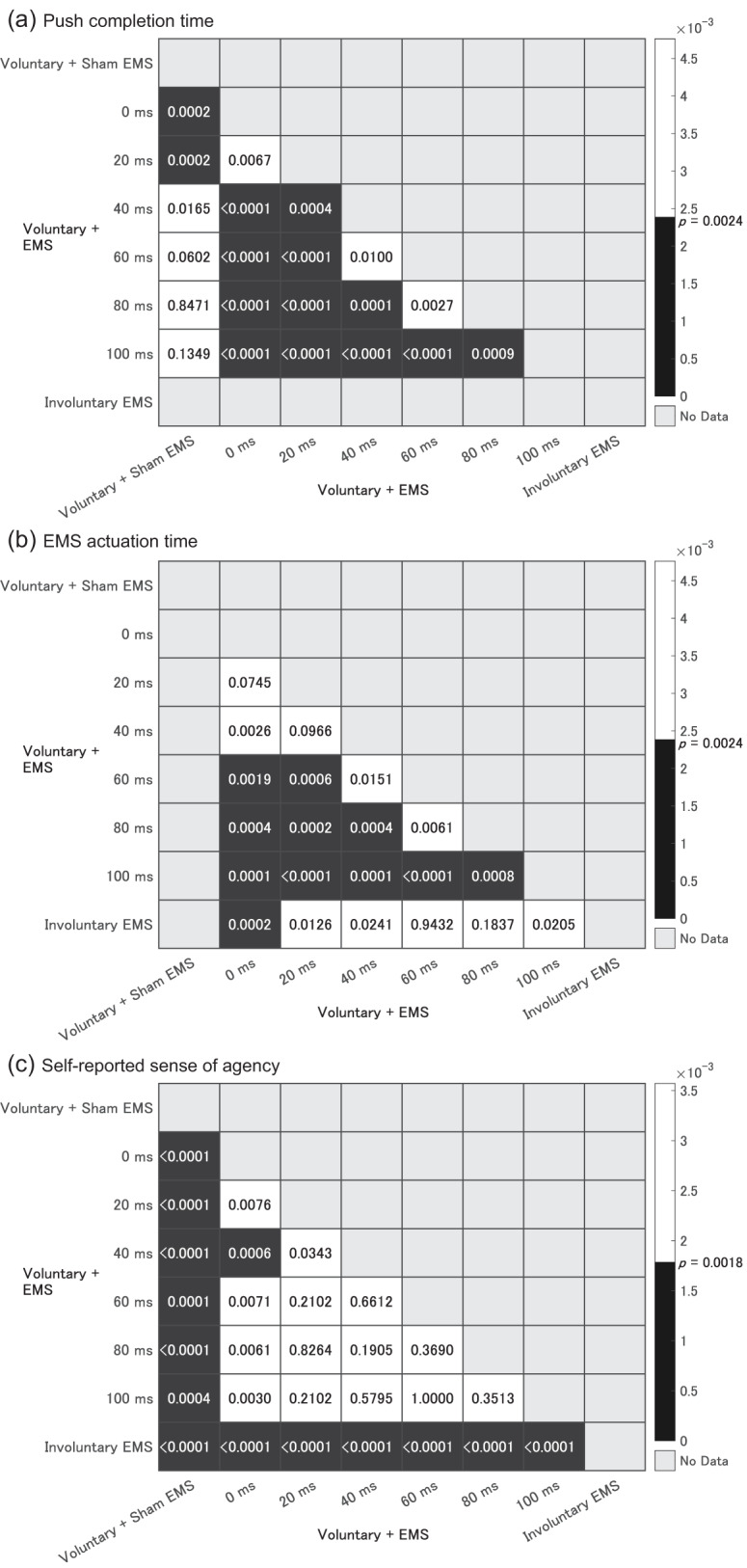
Heatmaps showing *p*-values of multiple comparisons of paired t-tests with Bonferroni correction. Combinations of conditions with significant differences are shaded in black. (A) Push completion time; (B) EMS actuation time; (C) Self-reported sense of agency.

We identified the EMS application timing that contributed to pushing acceleration among six EMS application timings. Because EMS is applied after the participant voluntarily releases the switch, the acceleration effect of EMS manifests as a reduction in the time from switch-off to switch-on, which is defined as the push completion time (solid line graph with triangle plots in the top graph of Fig. 5). Push completion times decreased as the EMS application time (dashed gray line in the top graph of Fig. 5) decreased. The Bonferroni-adjusted paired t-test revealed significant differences between *Voluntary + Sham EMS* and each of the 0-ms and 20-ms conditions ( $p = 0.0002$; Fig. 6A). The interval from EMS application time (dashed gray line in the top graph) to push completion time (solid black line) indicates the time from EMS application to switch pushing action, which is termed the EMS actuation time (shown as bar graphs in the top graph of Fig. 5). The EMS actuation time in only the 0-ms condition was significantly longer than that in the *Involuntary EMS* condition ( $p = 0.0002$; Fig. 6B). This suggests that the voluntary extension of the finger releasing the switch may have been counteracted by the flexion movement induced by EMS applied involuntarily simultaneously during switch release. The results imply that EMS applied under the 20-ms condition accelerated the pushing movement without interfering with voluntary movements.

Judgment errors and perceptual shifts are illustrated in Table 3 and the middle graph of Fig. 5. For each of the eight conditions, a paired t-test was employed to determine whether the perceptual shift was significant, similar to Experiment 1. Significant later shifts were observed in action task judgments only under the 20-ms condition in *Voluntary + EMS* ( $p = 0.0160$), while no significant shifts were observed in the other conditions (Table 3). Tone judgments exhibited significant early shifts under all conditions ( $p\; < \; 0.0001$). Only the 20-ms in *Voluntary + EMS* produced significant binding effects consistent with the single voluntary movements. Effect sizes, representing differences in means standardized independently of sample size, were not small (Cohen’s $d = 0.2834$ in action tasks and $d = 1.230$ in tone tasks), indicating significant shifts.

**Table 3:** Judgment error, perceptual shift, and paired t-test results between baseline and operant conditions in Experiment 2.

Condition		Judgment event	Mean judgment error (S.D.) (ms)		Mean perceptual shift (S.D.) (ms)
			Baseline	Operant	
*Voluntary + Sham EMS*		Action	12 (88)	35 (68)	23 (68), $t \,(16) = 1.377$; $p = 0.1876$
		Tone		−103 (87)	−123 (86), $t \,(16) = 5.928$; $p\; < \; 0.0001$
*Voluntary + EMS*	0 ms	Action	8 (82)	25 (74)	16 (42), $t \,(16) = 1.620$; $p = 0.1247$
		Tone		−106 (108)	−126 (81), $t \,(16) = 6.394$; $p\; < \; 0.0001$
	20 ms	Action	16 (72)	39 (87)	23 (35), $t \,(16) = 2.692$; $p = 0.0160$
		Tone		−79 (84)	−98 (70), $t \,(16) = 5.669$; $p\; < \; 0.0001$
	40 ms	Action	19 (83)	27 (74)	8 (58), $t \,(16) = 0.538$; $p = 0.5982$
		Tone		−85 (96)	−105 (61), $t \,(16) = 7.063$; $p\; < \; 0.0001$
	60 ms	Action	9 (67)	40 (93)	31 (69), $t \,(16) = 1.842$; $p = 0.0841$
		Tone		−97 (82)	−117 (76), $t \,(16) = 6.350$; $p\; < \; 0.0001$
	80 ms	Action	4 (127)	29 (80)	25 (115), $t \,(16) = 0.905$; $p = 0.3789$
		Tone		−89 (101)	−109 (81), $t \,(16) = 5.552$; $p\; < \; 0.0001$
	100 ms	Action	33 (54)	42 (56)	9 (34), $t \,(16) = 1.109$; $p = 0.2838$
		Tone		−74 (92)	−94 (96), $t \,(16) = 4.015$; $p\; < \; 0.0001$
*Involuntary EMS*		Action	44 (76)	56 (100)	12 (51), $t \,(16) = 0.947$; $p = 0.3575$
		Tone		−86 (95)	−106 (71), $t \,(16) = 6.110$; $p\; < \; 0.0001$
*Tone*		Tone	20 (77)		

The summary statistics for self-reported sense of agency are depicted in white box plots in the bottom graph of Fig. 5. Sense of agency in the *Voluntary + Sham EMS* condition significantly exceeded that observed in the six EMS application conditions ( $p = 0.0004$ in the 100-ms condition, $p\,\leqq\, 0.0001$ in the 0–80-ms conditions; Fig. 6C). Further, it was notably lower in the *Involuntary EMS* condition compared to that in the six conditions ( $p\; < \; 0.0001$; Fig. 6C). *Voluntary + EMS* conditions showed no significant differences in the sense of agency except between 0- and 40-ms conditions ( $p = 0.0006$). The median value of self-reported sense of agency ranged from 4 to 5, except for the 0-ms condition (median $= 3$), showing no specific trend. A lower explicit sense of agency under 0-ms condition is associated with significantly shorter EMS actuation time, suggesting interference between EMS-supported acceleration and voluntary movements. Although the explicit sense of agency under the 20-ms condition, wherein a significant binding effect was observed, was not as high as that in a single voluntary movement, median self-reported agency scores were more than three levels higher than under the *Involuntary EMS* condition, with 50% of trials scoring $\ge 4$ (maximum $= 6$).

In this experiment, participants judged when they or the EMS pushed the switch, establishing a causal relationship between all action tasks and subsequent tones. When voluntary movements of the proposed push method were combined with involuntary EMS movement, the voluntariness of the push movement was compromised if the push timing preceded the intended timing by the participant. Participants continued to push the switch without stopping the movement even when EMS interference occurred. This condition differs from scenarios where the participants are instructed to stop the movement when their movement is interrupted by the involuntary movement (Haggard & Clark, 2003) or the case wherein the participant inhibits the movement (Haggard, Poonian & Walsh, 2009). Thus, we investigated differences in movement intentionality across conditions when EMS elicited involuntary movements consistent with ongoing movement intentions at various application timings using 20-ms intervals.

Two measures of sense of agency suggested that intentionality was suppressed in nearly all six EMS application conditions. Action task judgments remained nonsignificantly shifted across most conditions, and self-reported sense of agency was lower compared to that in the *Voluntary + Sham EMS* condition. Meanwhile, the binding tendencies in the action and tone tasks differed from the unbinding effects of action (Haggard & Clark, 2003) and tone judgments (Haggard, Poonian & Walsh, 2009). These results indicate that when applied EMS interferes with or accelerates voluntary movement, movement intentionality is compromised but not inhibited. However, the significant shift in the tone task observed across all conditions should be considered to be the influence of the experimental design itself, given the findings of Experiment 1 (refer to the Discussion section for details).

Among the conditions involving the proposed voluntary push, only the 20-ms condition of *Voluntary + EMS* significantly bound the action and tone judgments together. These significant shifts mirrored the typical intentional binding observed in the single voluntary movement task of Experiment 1. Suppose the findings of Experiment 1 hold, where only intentional movements led to significant perceptual shifts in the action task. In that case, this result implies that movements under the 20-ms condition were intentional. However, the explicit sense of agency under the 20-ms condition, with a median value of 4, did not remain as high as under the other conditions. Additionally, there were no significant shifts in the action task under the *Voluntary + Sham EMS* condition despite the applied stimulus not contributing to the movement, indicating voluntary pushing. The significant shift observed in the action task under the 20-ms condition suggests a link to the retention of the sense of agency. However, definitive conclusions cannot be made owing to conflicting results.

In this experiment, we investigated perceptual shifts for a combination of the proposed voluntary and EMS-induced push movements, with a 20-ms offset interval between 0 and 100 ms. It was evident that only EMS applied 20 ms after switch release significantly shifted action judgments despite significantly accelerating the pushing movement. This finding aligned with the binding trend observed for single voluntary movements but contradicted with the results for single involuntary EMS movements. Analysis of the pushing time indicated that this application condition significantly accelerated the pushing movement without impeding voluntary movements. The analysis of measurement results indicates that the 20-ms condition in *Voluntary + EMS* can accelerate the pushing motion and maintain a sense of agency. However, the explicit sense of agency did not exhibit a specific increase under the 20-ms condition, contradicting the indication of a perceptual shift. In the subsequent section, we transitioned to a more generalized task, such as a reaction time task, to evaluate the effects of this EMS application condition.

## Experiment 3: evaluation of the ems application based on the specified timing

This experiment aimed to ascertain whether the EMS application timing determined in Experiment 2 accelerated users’ pushing while preserving their sense of agency. We incorporated a typical reaction time task in which the timing of movement was determined by visual stimuli, similar to a previous study by Kasahara, Nishida & Lopes (2019). This approach aimed to demonstrate the potential of the proposed EMS application method.

### Method

#### Participants

Ten healthy right-handed volunteers (all male; mean age = 22.5 years; S.D. = 1.4 years) participated in the experiment. The sample size was determined based on a study by Kasahara, Nishida & Lopes (2019). None of them had participated in Experiments 1 or 2. All participants were provided with written informed consent following the procedures approved by the Ethics Committee at the Osaka University Graduate School of Engineering Science (approval number: R4-12). All of them had previous experience with EMS.

#### Procedure

**Figure 7:**
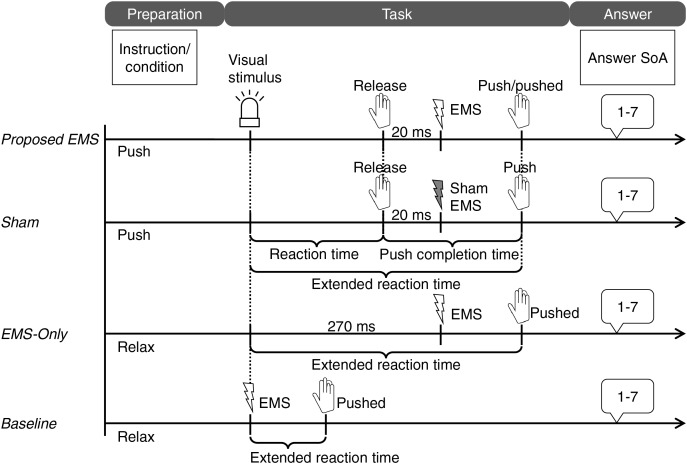
Tasks in Experiment 3. Image created in Microsoft PowerPoint. Icons taken from Microsoft PowerPoint.

This experiment involved reaction time tasks without the tone using the proposed voluntary push. These experimental tasks are shown in Fig. 7. The participants read the instructions (wait condition: with or without pushing the right-handed switch; task: after visual stimulus, switch pushing or relaxation) on the display and pushed the left-handed switch to start the trial. After the following four tasks, we asked the participants to assess their sense of agency on a seven-point Likert scale, like in Experiment 2.

***Proposed EMS*:** The participants were asked to perform the proposed voluntary push as fast as possible after the light-emitting diode (LED) came on. The EMS was applied 20 ms after the participants released the switch.

***Sham*:** The instructions to the participants were the same as those for the proposed task. The sham EMS was applied with a time offset (20 ms).

***EMS-Only*:** The participants were instructed to relax without activation, even when the LED came on. Assuming a reaction time of 250 ms from the visual stimulus until the finger released the switch (Hyman, 1953), the EMS was activated 20 ms later or 270 ms after the LED was turned on.

***Baseline*:** Only the EMS applied simultaneously with visual stimulation moved the finger. The participants were required to carry out the same tasks as in the *EMS-Only* condition. This condition assumes a typical EMS system in which the movement timing instruction and EMS initiation are simultaneously provided.

We informed them that electrical stimulation is applied in all conditions. Twenty trials per condition were randomly conducted.

The system from Experiment 1 or 2 was used for this experiment, with the clock and speaker removed. The parameters of the EMS and sham EMS were calibrated for each participant, similar to the case in the previous experiments. One participant could not flex the ring finger independently using EMS. Thus, the participant used the middle finger as an alternative.

### Results and discussion

The extended reaction time ranging from the visual stimulus to task completion was measured across all conditions (Fig. 7). Under the *Proposed EMS* and *Sham* conditions, the extended reaction time was longer than the traditional reaction time owing to the combination of two tasks: quickly releasing the switch based on visual stimulus and pushing the switch again in sequence. The mean time from visual stimulus to switch release, termed as the reaction time in this experiment, was 235.4 (S.D. = 17.69) ms, consistent with the result of a previous study (Hyman, 1953) where the reaction time from visual stimulus to switch pushing was 250 ms. Trials with reaction times exceeding 400 ms were excluded owing to participant distraction. For the two conditions involving voluntary movements, the *Proposed EMS* and *Sham* condition, the time between stimuli response (switch-off) and switch pushing was also measured, which was termed as the push completion time. It is worth noting that the EMS acceleration effect is directly linked to the push completion time because the EMS application was initiated only after participants voluntarily released the switch.

**Figure 8:**
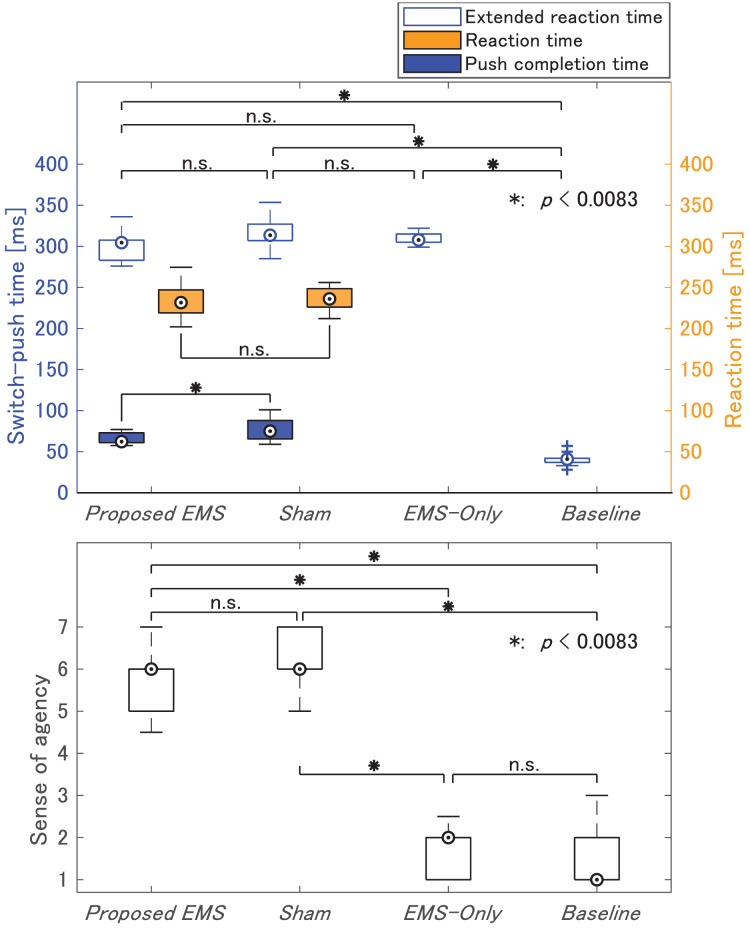
Median of the switch-push time and the self-reported sense of agency in Experiment 3.

Figure 8 shows box plots illustrating the median summary statistics of the extended reaction time, push completion time, reaction time (shown in the top graphs), and sense of agency (in the bottom graphs). Normality was not confirmed by Jarque–Bera tests. Therefore, a Bonferroni-adjusted Wilcoxon signed-rank test was conducted as a nonparametric test.

First, we compared the acceleration effect of pushing between the two application conditions, the *Proposed EMS* and *Sham* condition. Push completion times under the *Proposed EMS* condition were significantly shorter than those under the *Sham* condition ( $p = 0.0039$). This acceleration effect was consistent with the findings for the 20-ms condition of *Voluntary + EMS* in Experiment 2, where push completion times were significantly shorter than those under the *Voluntary + Sham EMS* condition. These results suggested that participants did not rely on feeling the electrical stimulation to accelerate their pushing actions.

Next, we focused on the self-reported sense of agency under the *Proposed EMS* condition, which successfully accelerated the pushing movement. Compared to conditions without voluntary movements, such as *EMS-Only* and *Baseline*, the sense of agency was significantly higher ( $p = 0.0020$) under the *Proposed EMS* condition. Further, we compared the *Proposed EMS* condition with the *Sham* condition to elucidate any loss of agency but found no significant differences ( $p = 0.1875$). Therefore, the explicit sense of agency in the proposed movement was not significantly diminished. This finding aligns with the results of Experiment 2, where a significant binding between action and tone was only observed under the 20-ms condition of *Voluntary + EMS*. However, the self-reported sense of agency in this experiment was higher than that under the same condition in Experiment 2 (see the Discussion section for details).

Through the reaction time task incorporated in this experiment, we demonstrated that it was feasible to significantly reduce the push completion time while maintaining self-reported sense of agency at a level comparable to the *Sham* condition. This finding is consistent with the results of Experiment 2, where the binding effect remained similar to that observed for single voluntary movements despite significantly reduced push completion time. This experimentally supports for the relation between the explicit sense of agency and the significant shift in action and tone judgments (the intentional binding effect) observed in the combined voluntary and EMS movements under the 20-ms condition.

## Discussion

When the human–computer integration technology, which combines autonomous humans and devices, extends human action skills, it is crucial to provide a sense of potential and continuous agency to the user to achieve acceptance by them (Moore, 2016; Cornelio et al., 2022). Building on a previous research that explicitly measured the sense of agency (Kasahara, Nishida & Lopes, 2019), our objective was to establish an appropriate EMS application timing capable of enhancing push acceleration and maintaining the sense of agency, guided by the intentional binding measure. Across three experiments, we confirmed that proposed voluntary movements and EMS-induced movements can be discerned based on the presence or absence of perceptual shifts in action tasks (Experiment 1), determined the EMS offset based on significant action binding (Experiment 2), and confirmed the efficacy of the proposed EMS application timing in maintaining the sense of agency and reducing the push time (Experiment 3).

### Perceptual shift of action and tone judgments in Experiments 1 and 2

The difference between involuntary EMS and proposed voluntary movements in this study was based on the presence of a significant shift in action judgment. Consistent with previous research (Strother, House & Obhi, 2010; Obhi & Hall, 2011; Dogge et al., 2012), a nonsignificant-later-shift tendency in action judgments during unintentional yet causally linked EMS pushing was replicated in Experiment 2. Conditions combining voluntary movement with EMS or sham EMS in Experiment 2 displayed a nonsignificant later shift in action judgments attributed to continuous participant movement (Haggard & Clark, 2003).

Conversely, the tone judgment revealed a significant and robust early shift across all conditions in both the experiments. Despite the sham EMS being a simple sensory event without causal relations or intentional movements, the action and tone tasks demonstrated a binding tendency (albeit not significant for the action). This contrasts with the minimal shifts or repulsion effects observed in two temporally contiguous sensory tasks (Haggard, Clark & Kalogeras, 2002; Buehner, 2015). Given that the tone binding effect is directly related to the prediction of the subsequent tone (Waszak, Cardoso-Leite & Hughes, 2012), the novel condition of this experiment—an abnormal electrical sensation—may have led participants to predict the timing of the tone. Another possible explanation is the influence of high-level beliefs about self-attribution and causality (Desantis, Roussel & Waszak, 2011; Dogge et al., 2012). This could be due to common instructions or environmental influences across the experiments. Before each task block, the experimenter explained all the tone and electrical stimuli, regardless of the task being judged. For instance, when judging the tone timing in the *Sham EMS* operant condition, the experimenter clarified that an electrical stimulus that did not induce movement would be applied at a time close to that of the tone. In addition, because the experimental environment was not soundproof, ambient noise may have reduced the reliability of the tones and increased the shift in tone judgments (Wolpe et al., 2013).

### Differences in explicit sense of agency between Experiments 2 and 3

In Experiment 3, we evaluated the effects of acceleration while maintaining a sense of agency through the reaction time task for the conditions of Experiment 2. Therefore, the *Proposed EMS* in Experiment 3 is the same condition as the 20-ms condition in *Voluntary + EMS* in Experiment 2; however, a limited sense of agency was reported in Experiment 2. The higher sense of agency in the movement accelerated by the EMS in Experiment 3 can be attributed to the different experimental environments of Experiments 2 and 3.

First, Experiment 2 involved multiple EMS application timings, while Experiment 3 employed only one EMS application time. Consequently, the EMS movement timing in Experiment 3 was more predictable, likely contributing to the self-attribution of movement (Frith, Blakemore & Wolpert, 2000; Blakemore, Wolpert & Frith, 2002). Second, the more desirable the task outcome, the higher the sense of agency based on the inferential process (Wegner, 2003; Sato & Yasuda, 2005; Yoshie & Haggard, 2013). The reaction time task in Experiment 3 required rapid pushing, and the voluntary push completion time in Experiment 3 (77.55 ms) was shorter than that in Experiment 2 (118.1 ms). Thus, participants in Experiment 3 may have actively attributed EMS-induced movements to themselves, recognizing these movements as aiding acceleration. The sense of agency explicitly reported in Experiment 3 likely depended on the integration of these two predictive and inferential cues (Moore, Wegner & Haggard, 2009).

### Comparison with previous studies using explicit measures of sense of agency

The availability of the proposed EMS-application time is revealed through a comparison with a previous study based on self-reports of sense of agency (Kasahara, Nishida & Lopes, 2019; Kasahara et al., 2021). We focused on the reported sense of agency because the EMS-application methods are different. The median sense of agency in Experiment 2 gradually increased from 3 to 5, depending on the delay in EMS application (as depicted in the bottom graph in Fig. 5). The authors of the previous study approximated the change in the sense of agency due to the EMS-application time as a sigmoid function and determined the optimal EMS timing at the midpoint with the highest slope. Since the EMS in this study was applied by triggering the voluntary switch-off movements of the participants themselves, it was not possible to apply it extremely earlier than the voluntary movements. Thus, the application scope in this experiment can be considered to correspond to the part of the sigmoid from around the midpoint to the latter half, where the increase rate decreases. Therefore, the established offset of 20 ms, at which the median sense of agency is 4, corresponds approximately to the vicinity of the midpoint. We suggest that it may be possible to find the EMS-application timing based on an implicit measure that is nearly equivalent to that based on an explicit measure of sense of agency, without the self-report of sense of agency.

### Advantage of the proposed method

Our proposed EMS-application method does not require individual adjustments and is robust to changes in environment and tasks, in contrast with the method in the previous study. The applicability of the method of pre-measuring each participant’s reaction time, as in the previous study, is limited because the user’s behavior varied depending on factors, such as the task difficulty, differences in individual motor strategies and abilities, and attention to each trial. The proposed application method, wherein the push action is designed so that a movement available as an EMS trigger occurs before each push, was adequately effective with different environments, users, and numbers of trials. Participants in the evaluation experiment (Experiment 3) exhibited a voluntary push completion time that was on average 33.00–40.57 ms shorter than the push completion times of the participants in the timing determination experiment (Experiment 2). Nevertheless, the *Proposed EMS* condition (employing EMS application timing established in Experiment 2 during the voluntary movement in Experiment 3) resulted in significantly faster movements compared to the voluntary control condition ( $p = 0.0039$).

### Limitation

Differences in the EMS effect due to the settings being adjusted for each participant may have affected the binding effect. Trials with EMS activation times of $> 200$ ms were eliminated in the binding measurement experiments because the EMS did not contribute to the pushing movement. However, the effect of EMS-induced movement is not constant due to changes in the user, electrode placement, and skin condition. Hence, quantitative data regarding the EMS effect (*e.g*., electromyogram or fingertip force induced by the EMS in personalized settings) are lacking.

## Conclusion

In this study, by measuring the intentional binding effect, we identified an EMS-application method that effectively accelerates pushing movement while also reducing the loss of the user’s agency. Our findings highlight the differences observed in the perceptual shift of action task judgments between involuntary EMS and voluntary movements. EMS applied 20 ms after switch release significantly shifted action judgments similar to voluntary movements, and contributed to maintaining the sense of agency in the reaction time task. The EMS-application time was determined without explicitly measuring the user’s sense of agency, and the EMS triggered by the users’ behaviors in a novel pushing setup was robustly effective against individual and environmental changes. Future studies should investigate whether the movement induced by the proposed EMS-application method is maintained after the EMS is removed, as is the case for self-reported sense of agency measures.

## Supplemental Information

10.7717/peerj.17977/supp-1 Supplemental Information 1 Raw measurements in Experiments 1, 2, and 3.

## Additional Information and Declarations

### Data Availability

The following information was supplied regarding data availability: The raw measurements are available in the Supplemental File.
